# Metabolic syndrome among psychiatric outpatients with mood and anxiety disorders

**DOI:** 10.1186/1471-244X-14-185

**Published:** 2014-06-21

**Authors:** Ching-I Hung, Chia-Yih Liu, Mei-Chun Hsiao, Nan-Wen Yu, Chun-Lin Chu

**Affiliations:** 1 Department of Psychiatry, Chang-Gung Memorial Hospital at Linkou, 5 Fu-Shing St, Kweishan, Taoyuan 333, Taiwan; 2 Chang-Gung University School of Medicine, Taoyuan, Taiwan

**Keywords:** Bipolar disorder, Depression, Anxiety, Metabolic syndrome

## Abstract

**Background:**

Few studies have simultaneously compared the impacts of pharmacotherapy and mental diagnoses on metabolic syndrome (MetS) among psychiatric outpatients with mood and anxiety disorders. This study aimed to investigate the impacts of pharmacotherapy and mental diagnoses on MetS and the prevalence of MetS among these patients.

**Methods:**

Two-hundred and twenty-nine outpatients (men/women = 85/144) were enrolled from 1147 outpatients with mood and anxiety disorders by systematic sampling. Psychiatric disorders and MetS were diagnosed using the Structured Clinical Interview for DSM-IV-TR and the new International Diabetics Federation definition, respectively. The numbers of antipsychotics, mood stabilizers, and antidepressants being taken were recorded. Logistic regression was used to investigate the impacts of pharmacotherapy and psychiatric diagnoses on MetS.

**Results:**

Among 229 subjects, 51 (22.3%) fulfilled the criteria for MetS. The prevalence of MetS was highest in the bipolar I disorder (46.7%) patients, followed by bipolar II disorder (25.0%), major depressive disorder (22.0%), anxiety-only disorders (16.7%), and no mood and/or anxiety disorders (14.3%). The percentages of MetS among the five categories were correlated with those of the patients being treated with antipsychotics and mood stabilizers. Use of antipsychotics and/or mood stabilizers independently predicted a higher risk of MetS after controlling for demographic variables and psychiatric diagnoses. When adding body mass index (BMI) as an independent variable in the regression model, BMI became the most significant factor to predict MetS.

**Conclusion:**

BMI was found to be an important factor related to MetS. Pharmacotherapy might be one of underlying causes of elevated BMI. The interactions among MetS, BMI, pharmacotherapy, and psychiatric diagnoses might need further research.

## Background

Metabolic syndrome (MetS) is characterized by several cardiovascular risk factors, including central obesity, hyperglycemia, dyslipidemia and hypertension, which are related to increased risks of cardiovascular diseases and type 2 diabetes mellitus [1,2]. The association between these metabolic disorders and the development of cardiovascular diseases might be multi-factorial, such as insulin resistance, oxidative stress, low-grade inflammation and vascular mal-adaptation [1,2]. Some studies have indicated an association between brain dysfunction and the pathogenesis of metabolic syndrome [3]. In recent years, many studies have investigated the prevalence of MetS among psychiatric patients. Most studies have focused on patients with schizophrenia or bipolar I disorder, and found that the two disorders carry a higher risk of MetS [4-8]. This may partially result from the fact that these patients are often treated with antipsychotics or mood stabilizers, some of which are associated with weight gain and abnormal lipid indices [8-10]. Moreover, other factors might also have an impact on MetS, such as lifestyle and shared features of hormonal, immunologic, and autonomic nervous system dysregulation between mental disorders and MetS [5,11,12].

Major depressive disorder (MDD) has been reported to be associated with MetS [13-17]. Some studies have demonstrated that depressive symptoms noted at baseline increased the risk of MetS at follow-up [13,17]. A shared pathophysiology has been reported between MDD and MetS [13], and some antidepressants are also related to weight gain [9,10]. In recent years, several new-generation antipsychotics have been used as an augmentation therapy for treatment-resistant MDD [18], which also increases the risk of MetS among MDD outpatients.

Compared with schizophrenia, bipolar I disorder and MDD, MetS among patients with anxiety disorders has received less attention. There are conflicting results regarding the relationship between MetS and anxiety symptoms or disorders. Some studies have reported a null finding regarding this relationship [19-21], while one study reported a positive finding between generalized anxiety disorder (GAD) and MetS [22]. Moreover, most studies have been concerned with the relationship between anxiety symptoms and MetS, and fewer studies have focused on the relationship between anxiety disorders and MetS [22,23].

Although many studies have reported the relationships between mental disorders and MetS, schizophrenia and bipolar I disorder have received more attention in this regard than MDD and anxiety disorders. To the best of our knowledge, no study has simultaneously investigated the prevalence of MetS in different mood and anxiety disorders and the impacts of mental diagnoses and pharmacotherapy on the risk of MetS among psychiatric outpatients with mood and anxiety disorders. Therefore, the aims of this study were to investigate the impacts of pharmacotherapy and mental diagnoses on the risk of MetS and the prevalence of MetS among psychiatric outpatients with mood and anxiety disorders. The above issues are important because understanding the impacts of mental disorders and pharmacotherapy on MetS provides evidence and strategies regarding how to prevent MetS. Moreover, investigating the prevalence of MetS in mood and anxiety disorders might help physicians to watch out for MetS in patients with mental disorders, who have a higher risk of MetS. We hypothesized that patients with bipolar I disorder might have the highest prevalence of MetS among these patients because patients with this disorder are usually treated with mood stabilizers and antipsychotics.

## Methods

### Subjects

The study was conducted from August 2008 to July 2009 in the psychiatric outpatient clinic of Chang Gung Memorial Hospital, a medical center in northern Taiwan, and was approved by the Institutional Review Board of the same hospital. The investigators screened the medical charts of psychiatric outpatients and excluded patients: 1) aged < 20 or > 60 years; 2) with psychotic disorders, such as schizophrenia, schizoaffective disorder, delusional disorder, and other psychotic disorders; 3) with mental retardation, delirium, dementia, and mental disorders due to general medical conditions. Patients with a mental disorder due to a general medical condition were excluded because patients in this group were heterogeneous and might be treated by a variety of medications. Therefore, the reasons for MetS among these patients are complicated and unclear. Patients with mood disorders with active psychotic symptoms or a current manic episode in the index month were also excluded because these patients may have difficulty completing self-administered scales or cooperating with the study process. The other outpatients were considered eligible subjects and were assigned a number. Systematic sampling was then used to draw one subject from 5 eligible subjects. Written informed consent, based on the guidelines regulated in the Declaration of Helsinki, was obtained from all subjects prior to study enrollment.

### Evaluation of mood and anxiety disorders

The Structured Clinical Interview for DSM-IV-text revision Axis I Disorders (SCID) [24,25] was used to diagnose bipolar disorders, MDD, and anxiety disorders, including panic disorder and/or agoraphobia, social phobia, simple phobia, obsessive-compulsive disorder and post-traumatic stress disorder and GAD. Patients with a lifetime history of MDD were divided into three states based on the severity of depression in the index month: a current major depressive episode (MDE), partial remission of a MDE, and full remission of a MDE. A current MDE was diagnosed based on the SCID and full remission was defined as a score on the Hamilton Depression Rating Scale (HAMD) ≤ 7 [26]. Bipolar disorders and MDD were commonly comorbid with anxiety disorders. If patients had mood and anxiety disorders simultaneously, their diagnoses were categorized as bipolar disorders or MDD, not anxiety disorders, because the impacts of major mood episodes on patients might be greater than those of anxiety symptoms, and prevention of these mood episodes is often the treatment focus. Therefore, patients were divided into bipolar I disorder, bipolar II disorder, MDD, anxiety-only disorders, and no mood and/or anxiety disorders.

### Evaluation of depression, anxiety, and somatic symptoms

In this study, three scales – the HAMD, the Depression and Somatic Symptoms Scale (DSSS), and the Hospital Anxiety and Depression Scale (HADS) – were used to evaluate the severities of depression, anxiety, and somatic symptoms [26-29]. The DSSS, a self-administered scale including 12 items for depression (Depression Subscale; DS) and 10 items for somatic symptoms (Somatic Subscale; SS), was developed to monitor both depressive and somatic symptoms [27,28]. Its reliability and validity have been reported in previous studies [27,28]. The HADS comprises 7 items for anxiety (HADS-A) and 7 items for depression (HADS-D) [29]. The total scores range from 0 to 52 for the HAMD, 0 to 36 for the DS, 0 to 30 for the SS, and 0 to 21 for both the HADS-D and HADS-A. A higher score indicates a higher severity of symptoms for all three scales.

### Evaluation of metabolic syndrome and related factors

MetS was diagnosed based on the new International Diabetics Federation definition [1], which includes central obesity (waist circumference ≥ 90 cm in men and ≥ 80 cm in women for Chinese subjects) or a body mass index (BMI) > 30 kg/m^2^ plus any two of the following four criteria: 1) elevated triglycerides (TG) (≥150 mg/dl or specific treatment for this lipid abnormality); 2) reduced high-density lipoprotein (HDL) cholesterol (<40 mg/dl in males; <50 mg/dl in females or specific treatment for this lipid abnormality); 3) elevated blood pressure (BP) (systolic BP ≥ 130 mmHg or diastolic BP ≥ 85 mmHg or treatment of previously-diagnosed hypertension); 4) elevated fasting plasma glucose (FPG) (≥100 mg/dl or previously-diagnosed type II diabetes).

An assistant investigator measured the body height and weight of subjects using standard instruments. BMI was then calculated. Some factors related to MetS were also recorded, such as alcohol dependence or abuse diagnosed based on the SCID, smoking habit, a regular exercise habit, pharmacotherapy factors, and other factors including parents’ history of diabetes mellitus, past history of psychiatric admission, and past history of attempted suicide. Antipsychotic, mood stabilizer, and antidepressant use were recorded, because the three psychotropics are commonly used in mood and anxiety disorders and have been reported to be related to MetS or weight gain [8-10]. The total numbers and costs of the three medication categories as well as the total years of psychiatric pharmacotherapy were calculated. Moreover, patients were requested to report their weight gain since accepting psychiatric pharmacotherapy (self-reported weight gain).

### Statistical analysis

**Figure 1:**
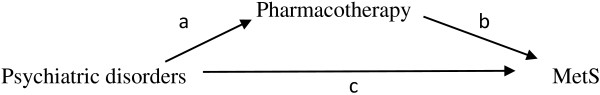
**The model of mediation analysis.** Note: The results of paths *b* and *c* are shown in the regression models I and II of Table 4, respectively. In path *a*, the relationship between the use of antipsychotics and/or mood stabilizers and bipolar I disorder was significant, but it was not possible to calculate the odds ratio because all patients with bipolar I disorder were treated with antipsychotics and/or mood stabilizers.

All statistical analyses were performed using SPSS for Windows 15.0. The independent *t* test, chi-square test, Pearson’s correlation, and Spearman’s correlation test were used where appropriate. In order to clarify the impacts of diagnosis and pharmacotherapy on MetS, Baron and Kenny’s procedures for mediation analyses, which were performed by multiple logistic regressions with Wald and forward selection, were used (Figure 1). In the first model (path c), the dependent variable was MetS and the independent variables were five demographic variables (age, gender, educational years, marital status, and employment) and psychiatric disorders (bipolar I disorder, bipolar II disorder, life-time history of MDD, any anxiety disorder, alcohol abuse or dependence). In the second model (path b), the dependent variable was MetS and the independent variables were five demographic variables and pharmacotherapy factors (years of pharmacotherapy, number of medications, use of antidepressants, use of mood stabilizers, use of antipsychotics, and use of antipsychotics and/or mood stabilizers). In the third model (path a), the dependent variable was the significant pharmacotherapy factor in the second model and the independent variables were five demographic variables and the significant psychiatric disorders in the first model. In the fourth model, the dependent variable was MetS and the independent variables were five demographic variables, the significant psychiatric disorders in the first model, the significant pharmacotherapy factor in the second model, and other factors (including parents’ history of diabetes mellitus, suicide attempt history, and history of psychiatric admission).

BMI was an important factor related to MetS and the first criterion of MetS. To understand whether psychiatric diagnoses and pharmacological factors are still significant after adding BMI as an independent factor, the fifth model was performed. The dependent variable was MetS and independent variables were BMI and all independent factors in the fourth model. In all statistical analyses, a two-tailed p value < 0.05 was considered statistically significant.

## Results

### Subjects

During the study period, a total of 2201 patients (880 males, 1321 females; mean age 46.6 ± 14.7 years) were screened and 1054 patients (479 males, 575 females; mean age 48.8 ± 17.9 years) were excluded due to the exclusion criteria (475 patients with psychotic disorders, 198 patients with dementia or aged > 60 years, 5 patients with a current manic episode, and 376 patients for other reasons, including 77 patients with mental retardation, 8 with delirium, 229 with mental disorders due to general medical conditions, and 62 aged < 20 years). Among the 1147 eligible subjects (401 males, 746 females; mean age 44.6 ± 10.4 years), 229 patients were enrolled by systematic sampling. Their scores in the three scales or their subscales, the indices of MetS, and the demographic variables are shown in Table 1. Although the indices for MetS were positively skewed and triglycerides and fasting plasma glucose had obvious leptokurtic distributions, these continuous variables were transformed to categorical variables for diagnosing MetS. Among the 229 subjects, 51 (22.3%) fulfilled the criteria for MetS. Compared with subjects without MetS, subjects with MetS were of significantly older age, had fewer years of education, took a greater number of medications with a higher cost, and had greater self-reported weight gain and higher indices of MetS. There were no significant differences in the scores of the three scales or subscales between subjects with and without MetS, with the exception of the SS score.

**Table 1:** **Indices of metabolic syndrome, scores of the three psychometric scales, and demographic variables in subjects with and without metabolic syndrome**^**†**^

		**Metabolic syndrome**	
	**Total sample (*n*** **= 229)**	**Yes (*n*** **= 51; 22.3%)**	**No (*n*** **= 178; 77.7%)**
Waist circumference (cm)	83.5 ± 10.0	94.5 ± 8.5**	80.4 ± 8.0
BMI	24.2 ± 4.0	28.3 ± 3.5**	23.1 ± 3.3
Triglycerides (mg/dL)	163.7 ± 205.3	305.9 ± 349.5**	122.9 ± 110.9
HDL (mg/dL)	54.1 ± 13.8	43.8 ± 9.5**	57.1 ± 13.4
Systolic BP (mmHg)	118.5 ± 161	129.3 ± 15.5**	115.4 ± 14.9
Diastolic BP (mmHg)	78.1 ± 10.8	85.0 ± 10.5**	76.1 ± 10.1
Fasting plasma glucose (mg/dL)	93.3 ± 19.5	107.1 ± 35.3**	89.4 ± 8.4
DS	13.8 ± 8.2	13.2 ± 7.5	13.9 ± 8.4
SS	10.2 ± 6.4	8.7 ± 4.7*	10.6 ± 6.7
HADS-D	8.3 ± 4.9	9.1 ± 5.3	8.1 ± 4.8
HADS-A	9.7 ± 4.7	9.0 ± 4.4	10.0 ± 4.7
HAMD	11.8 ± 6.9	11.7 ± 6.1	11.8 ± 7.1
Antipsychotics (Yes; %)	53 (23.1%)	18 (35.3%)*	35 (19.7%)
Mood stabilizers (Yes; %)	28 (12.2%)	10 (19.6%)	18 (10.1%)
Antidepressants (Yes; %)	205 (89.5%)	45 (88.2%)	160 (89.9%)
Number of medications	1.6 ± 0.8	1.8 ± 0.8*	1.5 ± 0.8
Medication cost per day (NTD)	47.8 ± 67.8	70.7 ± 87.4*	41.2 ± 59.7
Years of pharmacotherapy	4.4 ± 4.3	5.2 ± 5.1	4.1 ± 4.1
Self-reported weight gain (kg)	4.1 ± 6.7	7.5 ± 7.7**	3.1 ± 6.1
Age (years)	44.2 ± 9.9	47.3 ± 9.3*	43.3 ± 10.0
Years of education	11.4 ± 3.2	10.5 ± 3.1*	11.6 ± 3.3
Gender (Female)	144 (62.9%)	29 (56.9%)	115 (64.6%)
Paid employment (Yes)	139 (60.7%)	28 (54.9%)	111 (62.4%)
Married (Yes)	160 (69.6%)	35 (68.6%)	125 (70.2%)

Note: DSSS = Depression and Somatic Symptoms Scale; DS = depression subscale of the DSSS; SS = somatic subscale of the DSSS; HADS = Hospital Anxiety and Depression Scale; HADS-D = depression subscale of the HADS; HADS-A = anxiety subscale of the HADS; HAMD = Hamilton Depression Rating Scale; BMI = body mass index; HDL = high-density lipoprotein; NTD = new Taiwan Dollar (about 30 NTD = 1 US dollar).

*p < 0.05; **p < 0.01.

^†^Hypertension under treatment: *n* = 24; previously-diagnosed type II diabetes: *n* = 10; treatment for elevated TG: *n* = 10; treatment for reduced HDL: *n* = 10.

### Diagnosis and metabolic syndrome

Table 2 shows the prevalence of MetS with different diagnoses. Bipolar I disorder patients had the highest prevalence of MetS, followed by bipolar II disorder and MDD. Spearman’s correlation analysis showed that the percentages of MetS among the five categories were significantly correlated with the percentages of patients using antipsychotics (correlation coefficient *r* = 0.90, *p* = 0.04), those using mood stabilizers (*r* = 0.98, *p* < 0.01), those using antipsychotics and/or mood stabilizers (*r* = 0.90, *p* = 0.04), and the number of medications (*r* = 1, *p* < 0.001). However, the correlations with *p* = 0.04 became insignificant after using Bonferonni correction (significance should reach p < 0.0125) for multiple comparisons.

**Table 2:** **Percentages of fulfilled indices for metabolic syndrome, use of psychotropics, and number of medications among different diagnoses**^**†**^

	**Total sample (*n*** **= 229)**	**Bipolar I (*n*** **= 15)**	**Bipolar II (*n*** **= 16)**	**MDD (*n*** **= 141)**	**Only anxiety disorders (*n*** **= 36)**	**No mood and/or anxiety disorders (*n*** **= 21)**
MetS	22.3 (51)	46.7 (7)	25.0 (4)	22.0 (31)	16.7 (6)	14.3 (3)
Central obesity	46.3 (106)	73.3 (11)	62.5 (10)	45.5 (64)	41.7 (15)	28.6 (6)
BMI > 30 kg/m^2^	8.3 (19)	13.3 (2)	18.8 (3)	7.8 (11)	8.3 (3)	0 (0)
Elevated TG	35.8 (82)	60.0 (9)	37.5 (6)	36.9 (52)	30.6 (11)	19.0 (4)
Reduced HDL	30.6 (70)	46.7 (7)	37.5 (6)	29.8 (42)	27.8 (10)	23.8 (5)
Elevated BP	38.4 (88)	40.0 (6)	25.0 (4)	41.1 (58)	38.9 (14)	28.6 (6)
Elevated FPG	18.3 (42)	13.3 (2)	12.5 (2)	20.6 (29)	13.9 (5)	19.0 (4)
Antipsychotics	23.1 (53)	80.0 (12)	25.0 (4)	22.7 (32)	8.3 (3)	9.5 (2)
Mood stabilizers	12.2 (28)	93.3 (14)	31.3 (5)	6.4 (9)	0 (0)	0 (0)
Antipsychotics and/or mood stabilizers	27.9 (64)	100.0 (15)	50.0 (8)	25.5 (36)	8.3 (3)	9.5 (2)
Antidepressants	89.5 (205)	40.0 (6)	81.3 (13)	95.7 (135)	94.4 (34)	81.0 (17)
Number of medications	1.6 ± 0.8	2.4 ± 0.9	1.7 ± 1.1	1.6 ± 0.8	1.4 ± 0.6	1.1 ± 0.7

Note: MetS = Metabolic syndrome; BMI = body mass index; TG = triglycerides; Elevated BP = elevated systolic or diastolic blood pressure; FPG = fasting plasma glucose; HDL = high-density lipoprotein.

^†^Case numbers are shown in brackets.

Table 2 also shows the percentage of fulfilled MetS indices among the patients with different diagnoses. In the total sample, central obesity had the highest percentage, followed by elevated BP, elevated TG, reduced HDL, and elevated FPG. However, in the subjects with bipolar I or II disorder, the percentages of elevated TG and reduced HDL were higher than that of elevated BP.

Among 141 MDD patients, subjects with full remission (25.9%; 7/27) had the highest percentage of MetS, followed by those with a current MDE (24.4%; 10/41) and partial remission of MDE (19.2%; 14/73); however, the MetS percentages of the three states were not significantly different. There was also no significant correlation between the severity of depression, anxiety, or somatic symptoms (the HAMD, DS, SS, HADS-D, HADS-A scores) and the MetS indices (waist circumference, BMI, FPG, TG, BP, and HDL) among patients with MDD, except for a correlation of HADS-A (*r* = -0.25, *p* < 0.01) and HAMD (*r* = -0.21, *p* = 0.01) with waist circumference.

### Pharmacotherapy

Among 229 subjects, antidepressants were prescribed in 205 (89.5%) subjects. The most commonly-used antidepressant was paroxetine (percentage and mean dosage: 24.4%; 24.0 ± 11.1 mg), followed by trazodone (23.9%; 56.8 ± 30.6 mg), duloxetine (17.6%; 57.1 ± 24.8 mg), escitalopram (16.6%; 10.4 ± 4.7 mg), fluoxetine (12.2%; 36.0 ± 12.9 mg), and venlafaxine (10.2%; 128.6 ± 67.7 mg). Antipsychotics were prescribed in 53 (23.1%) subjects. Quetiapine (43.4%; 118.5 ± 100.3 mg) was the most commonly-used antipsychotic, followed by sulpiride (35.8%; 81.6 ± 41.5 mg) and olanzapine (11.3%; 5.8 ± 2.0 mg). Twenty-eight (12.2%) subjects were treated with mood stabilizers, including valproate (67.9%; 636.8 ± 305.9 mg), lamotrigine (17.9%; 200 ± 122.5 mg) and lithium (14.3%; 525.0 ± 150.0 mg).

Subjects being treated with antipsychotics had a higher risk of MetS than those who were free from treatment with antipsychotics (Table 3). Patients being treated with antipsychotics and/or mood stabilizers had a significantly higher risk of MetS (35.9% vs. 17.0%, Odds Ratio = 2.75, 95% CI = 1.43–5.27, *p* = 0.004) compared with patients who were not being treated with antipsychotics and/or mood stabilizers. There was no significant difference in the risk of MetS between those who were treated with antidepressants and those who were not.

**Table 3:** **Chi-square comparison of the percentages of patients with metabolic syndrome between groups**^**‡**^

	**Yes†**	**No**	***p***	**Odds ratio (95% CI)**
**Bipolar I**	**46.7 (7/15)**	**20.6 (44/214)**	**0.03**	**3.38 (1.16–9.23)**
Bipolar II	25.0 (4/16)	22.1 (47/213)	0.76	1.18 (0.36–3.82)
Major depressive disorder	22.0 (31/141)	22.7 (20/88)	1.00	0.96 (0.51–1.81)
Any anxiety disorders	18.4 (26/141)	28.4 (25/88)	0.10	0.57 (0.30–1.07)
Alcohol abuse or dependence	33.3 (14/42)	19.8 (37/187)	0.07	2.03 (0.97–4.23)
**Age > 45 years**	**29.7 (35/118)**	**14.4 (16/111)**	**< 0.01**	**2.50 (1.29–4.85)**
Exercise	26.5 (27/102)	18.9 (24/127)	0.20	1.55 (0.83–2.89)
Smoking	20.0 (12/60)	23.1 (39/169)	0.72	0.83 (0.40–1.72)
**Weight gain**	**29.3 (39/133)**	**12.5 (12/96)**	**< 0.01**	**2.90 (1.43–5.91)**
**Use of antipsychotics**	**34.0 (18/53)**	**18.8 (33/176)**	**0.02**	**2.23 (1.13–4.41)**
Use of mood stabilizers	35.7 (10/28)	20.4 (41/201)	0.09	2.17 (0.93–5.05)
Use of antidepressants	22.0 (45/205)	25.0 (6/24)	0.80	0.84 (0.32–2.25)
Parents’ history of diabetes mellitus	30.4 (21/69)	18.7 (30/160)	0.06	1.90 (0.99-3.63)
History of suicide attempt	25.7 (18/70)	20.8 (33/159)	0.49	1.32 (0.68-2.55)
**History of psychiatric admission**	**45.0 (9/20)**	**20.1 (42/209)**	**0.02**	**3.25 (1.27-8.36)**

^†^The percentage in the “yes” column represents the percentage of MetS in patients with a mental disorder (such as bipolar I disorder) or a variable (such as smoking). In the bracket, the numerator represents the case number of the patients with MetS.

^‡^Data with significant difference between the two groups are presented in boldface.

### Correlations of demographic variables, severity of depression, anxiety, and somatic symptoms with MetS indices

The correlations of the HAMD, DS, SS, HADS-A, and HADS-D with the MetS indices were not significant, with the exception of the correlation of HADS-A with waist circumference (*r* = -0.14, *p* = 0.04). Age was significantly correlated with FPG (*r* = 0.29, *p* < 0.001), TG (*r* = 0.16, *p* = 0.02), systolic BP (*r* = 0.31, *p* < 0.001), diastolic BP (*r* = 0.14, *p* = 0.04), BMI (*r* = 0.17, *p* = 0.01), and waist circumference (r = 0.23, *p* < 0.001). Educational years was correlated with FPG (r = - 0.26, *p* < 0.001), systolic BP (r = - 0.26, *p* < 0.001), BMI (*r* = - 0.22, *p* = 0.001), and waist circumference (*r* = -0.19, *p* = 0.003). Number of medications was correlated with TG (*r* = 0.19, *p* = 0.004), BMI (*r* = 0.20, *p* = 0.002) and waist circumference (*r* = 0.17, *p* = 0.01). Years of pharmacotherapy was not significantly correlated with the indices of MetS, with the exception of FPG (*r* = 0.14, *p* = 0.04).

### Other factors related to metabolic syndrome

Subjects with alcohol abuse or dependence had a tendency towards a higher MetS prevalence (33.3% vs. 19.8%, *p* = 0.07) as compared with those without (Table 3). There was no significant difference in the prevalence of MetS between subjects who exercised and those who did not and between subjects who smoked and those who did not. One-hundred and thirty-three subjects (58.1%) reported weight gain (8.2 ± 5.7 kg) after accepting psychiatric pharmacotherapy, 34 (14.8%) reported weight loss (4.6 ± 2.9 kg), and 62 (27.1%) reported no change in body weight. Compared with subjects without self-reported weight gain, the percentage of MetS was significantly higher in subjects with self-reported weight gain. Weight change (kg) was significant correlated with HDL (*r* = -0.2, *p* < 0.01) and diastolic BP (*r* = 0.18, *p* < 0.01). There was a trend (*p* = 0.06) that a father and/or mother with diabetes mellitus resulted in a higher risk of MetS. Subjects with a history of psychiatric admission had a significantly increased risk of MetS. A history of attempted suicide was not a significant factor in MetS.

### Factors independently predicting MetS

Table 4 shows the results of mediation analysis. In the first model, subjects of older age and those with bipolar I disorder had a higher risk of MetS. In the second model, use of antipsychotics and/or mood stabilizers, which exceeded the impact of an older age on the risk of MetS, could independently predict a higher risk of MetS and was the most significant factor independently predicting MetS. In the third model, there was a significant relationship between the use of antipsychotics and/or mood stabilizers and bipolar I disorder. However, it was not possible to calculate the odds ratio because all patients with bipolar I disorder were treated with antipsychotics and/or mood stabilizers (Table 2). The results of the fourth model, which were the same as the results of the second model, demonstrated that use of antipsychotics and/or mood stabilizers was a significant factor to predict MetS after controlling for psychiatric diagnoses and other factors.

**Table 4:** **Factors independently predicting metabolic syndrome among psychiatric outpatients with mood and anxiety disorders**^**‡**^

	**B**	**Wald**	**Odds ratio (95% CI)**	***p*-value**
Model I				
Age	0.05	6.65	1.05 (1.01–1.08)	0.01
Bipolar I disorder	1.31	5.48	3.71 (1.24–11.11)	0.02
Model II and IV^†^				
Age	0.05	7.97	1.05 (1.02–1.09)	0.005
Use of antipsychotics and/or mood stabilizers	1.15	11.08	3.17 (1.61–6.25)	0.001
Model V				
BMI	0.44	41.46	1.55 (1.36–1.77)	< 0.001
Age	0.05	4.51	1.05 (1.00–1.10)	0.04

^†^The results of models II and IV were the same.

^‡^The results shown in Table 4 were obtained by multiple logistic regressions with Wald and forward selection. In Model I, the dependent variable was MetS and the independent variables were five demographic variables and psychiatric disorders. In Model II, the dependent variable was MetS and the independent variables were five demographic variables and pharmacotherapy factors. In Model IV, the dependent variable was MetS and the independent variables were five demographic variables, the significant psychiatric disorders in the first model, the significant pharmacotherapy factor in the second model, and other factors (including parents’ history of diabetes mellitus, suicide attempt history, and history of psychiatric admission).

In the fifth model, BMI was the most significant factor in the prediction of MetS; however, the use of antipsychotics and/or mood stabilizers and bipolar I disorder was not significant.

## Discussion

After controlling for the demographic variables, the first regression model found that bipolar I disorder could predict MetS. The second regression model demonstrated that use of antipsychotics and/or mood stabilizers could predict MetS. Moreover, all patients with bipolar I disorder were treated with antipsychotics and/or mood stabilizers (Table 2). The fourth model found that use of antipsychotics and/or mood stabilizers could independently predict MetS after controlling for the demographic variables, psychiatric diagnoses, and other factors. Therefore, use of antipsychotics and/or mood stabilizers might have a mediation effect on the relationship of bipolar I disorder and MetS in this study. However, more evidence may be required to support this result. Previous studies have reported that some antipsychotics and mood stabilizers are related to an increase in the risk of lipid abnormality [30-32]. Future studies should consider that pharmacotherapy factors might have mediation effects on the relationship of psychiatric diagnoses and MetS. Bipolar I disorder had the highest risk of MetS in this study. This might partially result from the fact that patients with bipolar I disorder had the highest percentage of being treated with antipsychotics and/or mood stabilizers. Conversely, the prevalence of MetS among patients with only anxiety disorders (16.7%) or patients without any mood and anxiety disorders (14.3%) was close to the prevalence of MetS reported in a general population study in Taiwan (14.3%) [33]. Lower percentages of patients in the two groups were treated with antipsychotics (<10%) and none with mood stabilizers, and the number of medications was lower (Table 2). Moreover, the prevalence of MetS in the five groups was correlated with the percentage of patients taking antipsychotics and/or mood stabilizers as well as the number of medications. These results demonstrate that pharmacotherapy may have an important role in the etiology of MetS among these psychiatric outpatients.

In the fifth model, use of antipsychotics and/or mood stabilizers and bipolar I disorder was not significant after adding BMI as an independent variable. This might partially be due to BMI being the first criterion for MetS. Although this regression model found that BMI was the most significant factor for the prediction of MetS, this does not mean that pharmacological factors and psychiatric disorders are not important, because pharmacological factors and psychiatric disorders might be possible underlying causes of elevated BMI. Many previous studies have reported that some antipsychotics and mood stabilizers are associated with weight gain and abnormal lipid indices [8-10,30-32]. Moreover, some psychiatric disorders are related to changes in appetite and dieting behavior, unhealthy lifestyles, and dysfunctions of the endocrine system [8-12,34,35]. Future studies should further clarify the underlying causes of elevated BMI and the relationships between BMI, pharmacotherapy, and psychiatric disorders. In clinical practice, physicians should encourage patients to control BMI within an appropriate range.

Twenty-two percent of the MDD outpatients had MetS. This result was close to that of Richter’s report, which showed that 25% of depressive inpatients had MetS [36]. No significant difference was noted in the percentage of MetS between the three depressive states (MDE, partial remission, and full remission). There was also no significant correlation of the severity of depression, anxiety, or somatic symptoms with HDL, TG, FPG, or BP in the total sample and in patients with MDD. In fact, some studies have also demonstrated no significant correlation of depression and anxiety with MetS [19,20]. Two possibilities were assumed to explain the lack of a significant relationship between depressive states and MetS. First, the process of forming MetS might require a longer duration, such as several months or years; however, depressive severity might change within a shorter time, such as several days or weeks. Second, the impact of pharmacotherapy or other factors might be greater than that of depressive severity. However, these assumptions require more evidence.

There are several points worthy of note: 1) the prevalence of MetS in the total sample was higher (22.3% vs. 14.3%) than that reported in a general population study of MetS prevalence in Taiwan, diagnosed based on the IDF criteria [33]. Physicians should be aware of the risk of MetS when treating these outpatients. 2) The ranking of abnormal percentages of MetS indices in the total sample, MDD patients, and only anxiety disorder patients (Table 2) was similar to the results of an investigation into the prevalence of MetS in the general population of Taiwan, which demonstrated that the ranking of abnormal percentages was central obesity > elevated BP > elevated TG > reduced HDL > elevated FPG [33]. However, in the subjects with bipolar I and II disorder, the abnormal percentages of TG and HDL were higher than that of elevated BP in this study. Some previous studies have also shown that patients with bipolar I disorder have a higher percentage of elevated TG and reduced HDL than of elevated BP [4,37]. 3) Patients treated with antipsychotics had a higher risk of MetS (Table 3). The three most common antipsychotics in this study have been reported to be associated with increased abnormality of some MetS indices [30,38]. The dosage of antipsychotics in this study was lower than the suggested dosage for treating schizophrenia. This implied that even when using only a low dosage of antipsychotics, physicians still need to be aware of the indices of MetS. 4) Weight change was significantly correlated with HDL and diastolic BP. Moreover, the prevalence of MetS was significantly higher in subjects with self-reported weight gain post-pharmacotherapy. Physicians in clinical practice should educate patients about the clinical meaning of weight change and monitor it. 5) In univariate analysis, patients with MetS were treated with more medications (Table 1). One study reported that combination treatment of antipsychotics and mood stabilizers resulted in greater weight gain than mono-therapy with an antipsychotic or mood stabilizer among patients with bipolar I disorder [8]. Therefore, physicians should consider the impact of the synergistic effect of using multiple medications on MetS.

There are several methodological issues or limitations of this study to be addressed. 1) Because of limited budget for this study, selective sampling, which led to a small sample size within the disorder categories, was used. The small sample size hindered further analysis of the impact of each medication. Moreover, the small sample size meant that the findings of this study are not sufficiently robust and should be further examined in future studies. 2) This study was performed in a medical center and some exclusion criteria were established to prevent confounding factors. Bias might have been introduced during the enrollment process. 3) This study was a neutral clinical study and subjects might be treated with different medications and dosages. Only the three most common medications for mood and anxiety disorders were considered. Moreover, even in the same medication category, different medications carry different risks of MetS. 4) Although the sample sizes of patients with bipolar disorders and only anxiety disorders were small, this study simultaneously presented the prevalence of MetS among the five groups and demonstrated the impacts of diagnosis and pharmacotherapy. 5) The study only enrolled patients aged 20 to 60 years. Geriatric patients, who had a higher risk of MetS, were excluded. This might cause bias, and the percentage of MetS might be underestimated. 6) MetS in patients with schizophrenia was not investigated because this issue has been addressed previously in many studies. 7) The study did not include subjects without psychiatric disorders as a control group.

## Conclusion

BMI was the most important factor related to MetS, based on the fifth regression model. Pharmacotherapy might be one of underlying causes of elevated BMI. Based on the fourth regression model, pharmacotherapy was an important factor independently predicting MetS among psychiatric outpatients with mood and anxiety disorders. Subjects with bipolar I disorder had the highest prevalence of MetS among these patients. The percentages of MetS among the five groups were correlated with the percentages of patients being treated with antipsychotics and/or mood stabilizers. Among patients with MDD, the severity of depression, anxiety, and somatic symptoms were not significantly correlated with the indices of MetS. Patients with only anxiety disorders or without any mood or anxiety disorders, who had the lowest percentage of being treated with antipsychotics or mood stabilizers, had the lowest prevalence of MetS. However, explanation of the results should be tentative because of the small sample size of this study. These results should be further examined by future studies. Future studies of the risk of MetS should further explore the interaction between pharmacotherapy and mental diagnoses.

## Abbreviations

BMI: Body mass index; BP: Blood pressure; DSSS: Depression and somatic symptoms scale; DS: Depression subscale of the DSSS; FPG: Fasting plasma glucose; GAD: Generalized anxiety disorder; HADS: Hospital anxiety and depression scale; HADS-A: Anxiety subscale of the HADS; HADS-D: Depression subscale of the HADS; HAMD: Hamilton depression rating scale; HDL: High-density lipoprotein cholesterol; MDD: Major depressive disorder; MDE: Major depressive episode; MetS: Metabolic syndrome; SCID: Structured clinical interview; SS: Somatic subscale of the DSSS; TG: Triglycerides.

## Competing interests

All authors declare that they have no conflicts of interest.

## Authors' contributions

CIH and CYL designed the study and wrote the protocol. CIH, MCH, NWY, and CLC collected the data. CIH and MCH conducted the statistical analysis. CYL and CIH wrote the first draft of the manuscript. All authors contributed to and have approved the final manuscript.

## Pre-publication history

The pre-publication history for this paper can be accessed here:

http://www.biomedcentral.com/1471-244X/14/185/prepub
